# Knockdown of the TRPM4 channel alters cardiac electrophysiology and hemodynamics in a sex‐ and age‐dependent manner in mice

**DOI:** 10.14814/phy2.15783

**Published:** 2023-08-21

**Authors:** Prakash Arullampalam, Maria C. Essers, Mey Boukenna, Sabrina Guichard, Jean‐Sébastien Rougier, Hugues Abriel

**Affiliations:** ^1^ Institute of Biochemistry and Molecular Medicine, and Swiss National Centre of Competence in Research (NCCR) TransCure, University of Bern Bern Switzerland

**Keywords:** cardiac physiology, murine knockdown model, TRPM4 channels

## Abstract

TRPM4 is a calcium‐activated, voltage‐modulated, nonselective ion channel widely expressed in various cells and tissues. TRPM4 regulates the influx of sodium ions, thus playing a role in regulating the membrane potential. In the heart, TRPM4 is expressed in both cardiomyocytes and cells of the conductive pathways. Clinical studies have linked *TRPM4* mutations to several cardiac disorders. While data from experimental studies have demonstrated TRPM4's functional significance in cardiac physiology, its exact roles in the heart have remained unclear. In this study, we investigated the role of TRPM4 in cardiac physiology in a newly generated *Trpm4* knockdown mouse model. Male and female *Trpm4* knockdown (*Trpm4* ^−/−^) and wild‐type mice of different ages (5‐ to 12‐ week‐old (young) and 24‐week‐old or more (adult)) were characterized using a multimodal approach, encompassing surface electrocardiograms (ECG), echocardiography recordings, ex vivo ECGs in isolated heart, endocardial mappings, Western blots, and mRNA quantifications. The assessment of cardiac electrophysiology by surface ECGs revealed no significant differences between wild‐type and *Trpm4* ^−/−^ young (5‐ to 12‐week‐old) mice of either sex. Above 24 weeks of age, adult male *Trpm4* ^−/−^ mice showed reduced heart rate and increased heart rate variability. Echocardiography revealed that only adult male *Trpm4* ^−/−^ mice exhibited slight left ventricular hypertrophic alterations compared to controls, illustrated by alterations of the mitral valve pressure halftime, the mitral valve E/A ratio, the isovolumetric relaxation time, and the mitral valve deceleration. In addition, an assessment of the right ventricular systolic function by scanning the pulmonary valve highlighted an alteration in pulmonary valve peak velocity and pressure in adult male *Trpm4* ^−/−^ mice. Endocardial mapping recordings showed that applying 5 μM of the new TRPM4 inhibitor NBA triggered a third‐degree atrioventricular block on 40% of wild‐type hearts. These results confirm the key role of TRPM4 in the proper structure and electrical function of the heart. It also reveals differences between male and female animals that have never been reported. In addition, the investigation of the effects of NBA on heart function confirms the role of TRPM4 in atrioventricular conduction.

## 1 INTRODUCTION

Transient receptor potential melastatin‐related 4 (TRPM4) is a nonselective, cation‐permeable channel modulated by transmembrane voltage (Ehara et al., 1988; Launay et al., 2002; Nilius et al., 2003). It is encoded by the mouse *Trpm4* and human *TRPM4* genes, found on chromosome 7 in mice and chromosome 19 in humans, respectively (Guinamard et al., 2010; Launay et al., 2002; Nilius et al., 2004). TRPM4 channels are widely expressed in various cells and organs, including pancreatic β‐islet cells, neurons, immune cells, smooth muscle cells in blood vessels, prostate, bladder, and heart (Demion et al., 2007; Kruse et al., 2009; Partridge & Swandulla, 1988; Siemen, 1993).

Its conductance was first described in 1981 by Colquhoun et al. in rat cardiomyocytes (Colquhoun et al., 1981). TRPM4 is permeable for monovalent cations Na^+^ > K^+^ > > Cs^+^ > Li^+^ and is activated by intracellular Ca^2+^ (Launay et al., 2002; Nilius et al., 2003). Allowing the influx of sodium ions, TRPM4 activation depolarizes the cell membrane (Nilius & Flockerzi, 2014). The activation of TRPM4 is modulated by PKC phosphorylation, calmodulin, and PIP₂ (Chu & Stefani, 1991; Nilius et al., 2005). Intracellular nucleotides such as ATP, ADP, AMP, and the nonhydrolyzable ATP analog adenylyl‐imidodiphosphate (AMP‐PNP) have been shown to inhibit the activity of the TRPM4 channel (Nilius et al., 2004).

TRPM4 dysfunction has been linked to several cardiac conduction disorders. In 2009, a genetic study linked a human *TRPM4* mutation with cardiac bundle branch block (Kruse et al., 2009). Since then, several clinical and experimental studies have linked *TRPM4* mutations with other conduction disorders such as Brugada syndrome, atrioventricular block, and right bundle branch block (Liu et al., 2010, 2013; Stallmeyer et al., 2012). Interestingly, gain‐ and loss‐of‐function mutations have been suggested to lead to similar cardiac dysfunctions (Abriel et al., 2012; Demion et al., 2014). Experimental mouse studies using *Trpm4* ^−/−^ atrial cardiomyocytes not only showed that the cardiac action potentials duration was 20% shorter than their wild‐type counterparts but also that the sinus rhythm was altered (Hof et al., 2013; Simard et al., 2013). Currents from a nonselective cation channel with characteristics of TRPM4 currents have also been detected in murine sinoatrial nodes and human atrial cells (Demion et al., 2007; Guinamard et al., 2004). Despite this growing body of evidence illustrating the importance of TRPM4 in cardiac conduction, the role of TRPM4 in human and murine cardiac physiology remains to be determined.

Two main experimental approaches have been used to study TRPM4 function: TRPM4 inhibitors or *Trpm4* ^−/−^ mouse models. Both, however, have substantial limitations. Regarding the first approach, TRPM4 inhibitors for experimental use include flufenamic acid and the widely used drug 9‐phenanthrol (Guinamard et al., 2014). However, their specificity is disputed. Recent papers have shown that 9‐phenanthrol also inhibits the calcium‐activated potassium channel K_Ca_3.1 and the calcium‐activated chloride channel TMEM 16A, while flufenamic acid also inhibits calcium‐activated chloride channels (Burris et al., 2015; Garland et al., 2015; Gwanyanya et al., 2010). To circumvent this problem, we recently identified two new specific TRPM4 inhibitors in the frame of the NCCR TransCure consortium: 4‐chloro‐ 2‐[2‐(2‐chloro‐phenoxy)‐acetylamino]‐benzoic acid (CBA) and 4‐chloro‐2‐(2‐(naphthalene‐1‐ yloxy) acetamido) benzoic acid (NBA) (Arullampalam et al., 2021; Ozhathil et al., 2018). Although both CBA and NBA decrease the current mediated by human TRPM4 overexpressed in heterologous expression systems, surprisingly, only the NBA compound inhibits mouse TRPM4 currents in this system (Arullampalam et al., 2021). These findings have yet to be confirmed in in vivo or ex vivo experiments. The second approach consists of *Trpm4* ^−/−^ mouse models. Other groups have already developed and characterized such models, but the findings of those studies differed significantly (Demion et al., 2014; Mathar et al., 2010; Vandewiele et al., 2022). On the one hand, Vandewiele et al. and Mathar et al. noted no functional change between male *Trpm4* ^−/−^ and wild‐type mice under basal conditions (cardiac output, stroke work, preload, and ECG parameters) (Mathar et al., 2014; Vandewiele et al., 2022). On the other hand, Demion et al. found that male *Trpm4* ^−/−^ mice developed left ventricle hypertrophy, multilevel conduction blocks, increased ectopic activity, and ECG alterations (Demion et al., 2014). Although these observations are important to understand the potential roles of TRPM4 for cardiac function, they also illustrate the high level of complexity intrinsic to investigating the cardiac role of TRPM4 in a physiological environment. Possible explanations for the discrepancies are the different mouse strains and substrain used (129/SvJ for Mathar et al. C57BL/6N for Vandewiele et al. and, C57BL/6J for Demion et al.) as reported by Rebekka et al. (Medert et al., 2020).

To address these discrepancies, we generated a fourth *Trpm4* knockdown mouse model, which differs from the model used by Demion et al. only in its substrain C57BL/6JRj background (Demion et al., 2014). C57BL/6 background mice have been chosen based on two criteria: (1) Male and female mice from this strain are known to be good breeders and (2) this strain is commonly used for transgenic animal production. We decided to use the substrain “RJj” for three reasons: (1) C57BL/6JRj mice, in contrary to the 129 background animals, present an RR interval more stable over the life span of the animals, a parameter altered in *Trpm4* knockdown mouse models (Vignier et al., 2019), (2) those mice are protected from overweigh which can influence, if append, the interpretation of the data (Siersbaek et al., 2020) and (3) no cardiac hypertrophy has been, till today, descript concerning thus substrain in contrary to the C57BL/6N (Kiper et al., 2013; Moreth et al., 2014). Moreover, the C57BL/6JRj strain has the advantage of being well characterized and readily available via Jackson laboratory (https://janvier‐labs.com/fiche_produit/2‐c57bl‐6jrj/), enabling us to perform backcrossing with the mouse model to achieve a genetically homogenous “pure” background. With this new *Trpm4* ^−/−^ mouse model, we first investigated whether we could confirm the previous observations done by the other groups on the role of TRPM4 in cardiac physiology (Demion et al., 2014; Mathar et al., 2010, 2014; Vandewiele et al., 2022). Secondly, we assessed the influence of the age and sex of the animals on TRPM4 function. Lastly, we tested the new promising human and mouse TRPM4 inhibitor NBA in a mouse model for the first time.

## 2 METHODS

### 2.1 *Trpm4* ^−/−^ C57BL/6JRj mouse model

A new *Trpm4* ^−/−^ mouse model has been generated for this research project. The *Trpm4* ^−/−^ (B6.Cg‐ Trpm4tm1.2‐PG) mouse model is a global knockdown of the *Trpm4* gene by excision of exons 10. Mice were backcrossed on a C57BL/6JRj background (Janvier) (Figure S1). To fulfill the “3R” criteria (*Reduce*, *Reuse*, and *Refine)*, male and female *Trpm4* ^−/−^ mice and wild‐type animals at different matched ages were used for all experiments. These included young (5‐ to 12‐week‐old) and adult (more than 24‐week‐old) animals. Mice were housed in a controlled, pathogen‐free, environment (21 ± 1°C; humidity 60%; lights on 08:00 am–08:00 pm; food and water available ad libitum; enriched environment) with maximum five mice per cage. According to the Swiss Federal Animal Protection Law, all animal experiments were performed and approved by Bern's Cantonal Veterinary Administration (license BE88 2022). This investigation conforms to the Guideline for the Care and Use of Laboratory Animals, published by the US National Institutes of Health (NIH publication no. 85–23, revised 1996).

### 2.2 Western blot

The expression of the TRPM4 channel was assessed in whole‐cell lysates. First, heart tissue was lysed for 1 h at 4°C in lysis buffer (50 mM HEPES (Sigma‐Aldrich Chemie GmbH, Taufkirchan; 54457) pH 7.4, 1.5 mM MgCl_2_ (Sigma‐Aldrich Chemie GmbH, Taufkirchan; 63069), 150 mM NaCl (Merck KGaA, Darmstadt, Germany; S3014), 1 mM EGTA (Sigma‐Aldrich Chemie GmbH, Taufkirchan; 03779) pH 8, 10% glycerol (Sigma‐Aldrich Chemie GmbH, Taufkirchan; 49770), 1% Triton X‐100 (Sigma‐Aldrich Chemie GmbH, Taufkirchan; 93420), and Complete® protease inhibitor cocktail (Roche Diagnostics, Mannheim, Germany; 11873580001). Then it was centrifuged at 4°C, 16′000 *g* for 15 min, and the pellet was discarded. The protein concentration of each of the lysate samples was measured in triplicate by Bradford (Interchim, Montluçon FRANCE; UPF686420) assay and interpolated by a bovine serum albumin (BSA) (Interchim, Montluçon FRANCE; UP36859A) standard curve. Samples were denatured at 95°C for 5 min before loading them on a gel. Sixty μg of protein for each sample was run at 150 V for 1 h on 9% polyacrylamide (Applichem GmbH, Darmstadt, Germany; A4983.0250) gels. The Turbo Blot dry blot system (Biorad, Hercules, CA, USA) was used to transfer the samples to a nitrocellulose membrane. All membranes were stained with Ponceau as a qualitative check for equivalent loading of total protein. Membranes were then rinsed twice with PBS 1X (Sigma‐Aldrich Chemie GmbH, Taufkirchan; 10,010–015) before using the SNAP i.d. system (Millipore, Zug, Switzerland) for Western blotting. The membrane was blocked with 0.1% BSA in PBS 1X for 10 min. The membranes were incubated for 10 min with rabbit primary anti‐mouse TRPM4 (**Q7TN37–1)** antibody (epitope: _2_VGPEKEQSWIPKIFRKKVC_10_) (generated by Pineda, Berlin, Germany) diluted 1:750 in PBS 1X + 0.1% Tween‐20 (Chemsolute, Aesch, Switzerland; 80221000) using the SNAP i.d. system (Millipore, Billerica, MA, USA). Membranes were subsequently washed four times in PBS 1X + 0.1% Tween‐20 before incubating with fluorescent secondary antibodies. Secondary antibodies (IR Dye 800 CW, 1:1000 in PBS 1X/Tween‐20, LI‐COR Biosciences, Lincoln, NE, USA) were added for 10 min. After four more washes with PBS 1X (+ 0.1% Tween‐20 and 3 washes in PBS 1X, membranes were scanned with the Odyssey® Infrared Imaging System (LI‐COR Biosciences, Bad Homberg, Germany) for detection of fluorescent protein. Subsequent quantitative analysis of protein content was achieved by measuring and comparing band densities (equivalent to fluorescence intensities of the bands) using Odyssey software version 3.0.21

### 2.3 Enzyme‐linked immunosorbent assay kit *(ELISA)*

*Left ventricular blood samples were extracted by performing a cardiac punction from anesthetized (ketamine/xylazine, intraperitoneal; one time; 200/20 mg/kg body weight) **(*** Streuli, Uznach, Switzerland; 7680538150144 and Vetoquinol, Lure, France: 47190029) *adult male and female (wild‐type and Trpm4* ^−/−^ *) mice using 25‐gage needle (Braun, Sempach, Switzerland). After extraction, the blood was left to clot for approx*. Sixty *minutes at room temperature, before the samples were spun‐down at 4°C, 1*′*000 g for 20 min to separate the sera*. The sera were snap frozen and stored at −80°C for less than 2 months. The Mouse N‐terminal Pro‐Brain Natriuretic Peptide (NT‐ProBNP) ELISA kit MBS2023403 (MyBiosource, San Diego, CA, USA) was used following the manufacturer's instructions. The NT‐proBNP concentrations have been quantified at 450 nm using the GloMax® Explorer Multimode Microplate Reader (Promega, Dubendorf, Switzerland).

### 2.4 Surface electrocardiograms

Three‐lead surface electrocardiograms (ECG) were recorded during 1 min in 5‐, 6‐, 7‐, 8‐, 9‐, 11‐, 12‐, and above 24‐week‐old mice under anesthesia (isoflurane IsofloH, ABBOTT S.A, Madrid, Spain: induction: 2.0 vol.% in 1000 cm^3^ O_2_/minute; maintenance: 1.5 vol.% in 500 cm^3^ O_2_/minute). Body temperature was maintained at 37°C using a thermic pad. Data were collected and transmitted to a computer via an analog‐digital converter (National Instruments, Austin, TX, USA). Power lab bio‐amplifiers collected the ECG tracings, which were low‐ and high‐pass filtered (at 200 Hz and 0.1 Hz, respectively) and sampled at a speed of 1 k/s. Data from lead II configuration were analyzed offline by LabChart7 Pro (AD Instruments, Castle Hill, NSW, Australia). Firstly, the ECG tracing was scanned for arrhythmias and noise. Heart rate (HR), P duration, RR, PR, and QRS intervals were determined by analyzing three stable sequences of 30 s for each ECG. Heart rate variability (HRV) was quantified using the root mean square of successive differences between normal heartbeats (RMSSD). RMSSD is obtained by first calculating each successive time difference between heartbeats. Then, each of the values is squared and the result is averaged before the square root of the total is obtained. The same time duration has been used for each group.

### 2.5 Echocardiography

Each anesthetized mouse (isoflurane IsofloH, ABBOTT S.A, Madrid, Spain; induction: 3.5 vol.% in 1000 cm^3^ O_2_/minute; maintenance: 1–1.5 vol.% in 1000 cm^3^ O_2_/minute) was placed in supine position on a heated table and secured to the table via 4 ECG leads attached to the mouse's limbs. Hair‐removing cream (Veet, Reckitt Benckiser, Granollers, Spain) was used to remove chest hair, and ultrasound gel (Quick Eco‐Gel, Lessa, Barcelona, Spain) was applied to enhance image quality. Echocardiography studies were done at baseline, using a Vevo 2100 ultrasound system (Visual Sonics, Toronto, Canada) equipped with a real‐time micro‐visualization scan head probe (MS‐550D) at around 740 frames per second. The HR and respiratory rates were monitored during the study. To assess the function of the heart, left ventricle (LV) systolic, LV diastolic, and right ventricle (RV) systolic function were assessed as follows. LV systolic function was assessed via the stroke volume, the ejection fraction, the cardiac output, the left ventricle mass corrected, and the fractional shortening parameters calculated from M‐mode measurement. The M‐mode cursor was positioned vertically to obtain a transthoracic parasternal short‐axis view. This classical LV M‐mode tracing approach allowed the visualization of both papillary muscles. The functional parameters of the heart were calculated using the following equations based on LV diameter measurements (adapted from American Society of Echocardiography guidelines and the Vevo 2100 protocol‐ based measurements and calculations guide):

Left ventricular end‐diastolic volume (LVEDV) (μL):

$$\text{LVEDV}=\left(7/\left(2.4+\text{LVEDD}\right)\right)*{\text{LVEDD}}^{3}$$

Left ventricular end‐systolic volume (LVESV) (μL):

$$\text{LVESV}=\left(7/\left(2.4+\text{LVESD}\right)\right)*{\text{LVESD}}^{3}$$

Stroke volume (SV) (μL):

SV=LVEDV−VESV.

Cardiac output (CO) (mL/minute):

$$\mathrm{CO}=V^{*}\mathrm{HR}.$$

HR: heart rate (beats per minute (bpm)).

Fractional shortening (%):

$$\mathrm{FS}=\left(\left(\text{LVEDD}–\text{LVESD}\right)*100\right)/\text{LVEDD}$$

LVEDD, left ventricle internal dimensions at end diastole diameter (cm); LVESD, left ventricle internal dimensions at end systole diameter (cm).

Left ventricular ejection fraction (LVEF) (%):

$$\text{LVEF}=\left(\left(\text{LVEDV}-\text{LVESV}\right)*100\right)/\text{LVEDV}$$

LVEDV, left ventricle end‐diastolic volume (μL); LVESV, left ventricle end‐systolic volume (μL).

Left ventricle mass corrected (LV mass corrected) (mg):

$$\begin{array}{c} \mathrm{LV}\;\text{mass corrected}\\ =\left(\left(1.053^{*}\left({\left(\text{LVEDD}+\text{LVPWED}+\text{IVSED}\right)}^{3}-{\text{LVEDD}}^{3}\right)\right)*0.8\right) \end{array}$$

LVPW, ED: left ventricle posterior wall, end diastole (cm).

IVS, ED: interventricular septum thickness at the end systole (cm). For the left ventricle mass corrected equation, the 1.053 g/mL value corresponds to the estimated density of the ventricle (Vinnakota & Bassingthwaighte, 2004). The factor 0.8 is a “correction” factor applied based on initial recommendations by the American Society of Echocardiography due to the overestimation in echocardiography measurements. The left ventricle diastolic function was assessed using pulse wave Doppler imaging of transmitral inflow. This was measured from the LV apical four‐chamber views. The transducer was angled so that the ultrasound waves would be parallel to the blood flow. Velocities of peak E wave (early filling wave) and peak A wave (late atrial contraction wave), mitral valve E/A ratio (MV E/A), deceleration time (MV decel), left ventricle isovolumetric relaxation time (IVRT), and mitral valve pressure halftime (MV PHT) were calculated based on the Doppler graph.

Mitral valve E/A ratio (MV E/A): MV E/A: ratio between velocities of peak E wave (early filling wave) and peak A wave (late atrial contraction wave).

Mitral valve pressure halftime (MV PHT) (simplified) (ms):

$$\mathrm{MV}\;\mathrm{PHT}={\mathrm{TV}}_{\max}/1.4-{\mathrm{TV}}_{\max}$$

TV_max_ corresponds to the time from the V_max_ velocity.

Mitral valve deceleration time (MV decel) (mm/s^2^): MV deceleration time corresponds to the time point from the V_max_ to the time point where the velocity is equal to zero.

Left ventricle isovolumetric relaxation time (IVRT) (ms): The isovolumic relaxation time is the time interval between the end of aortic ejection and the beginning of ventricular filling.

The right ventricle systolic function was also assessed using the pulmonary artery view via the pulse wave Doppler imaging of transpulmonary valve inflow. The Doppler graph was used to measure the peak velocity of blood flow at the pulmonary valve (PV peak vel), peak pressure (PV peak pressure), diameter (PV diam), and mean pulmonary artery pressure (mPAP).

Pulmonary valve peak velocity (PV peak vel) (mm/s): Corresponds to the interval between the onset of flow and peak flow.

Pulmonary valve diameter (PV diam) (mm): Corresponds to the diameter of the pulmonary valve.

Pulmonary valve peak pressure (PV peak pressure) (mm Hg): Corresponds to the maximum pressure at the level of the pulmonary artery.

Mean pulmonary artery pressure‐mPAP (mm Hg): mPAP = 90‐(0.62*AT_RVOT_).

AT_RVOT_ corresponds to the acceleration time of the right ventricular outflow tract measured from the beginning of the flow to the peak flow velocity.

### 2.6 Ex vivo ECG in isolated heart and endocardial mapping

Anesthetized mice (ketamine (200 mg/kg) and xylazine (20 mg/kg)) were sacrificed by cervical dislocation. Hearts were rapidly excised and retrogradely perfused using the Langendorff system (modified Krebs–Henseleit buffer (KHB) containing the following of mmol/L:116.5 NaCl_2_, 25 NaHCO_3_ (Sigma‐Aldrich Chemie GmbH, Taufkirchan; 792519), 4.7 KCl (Sigma‐Aldrich Chemie GmbH, Taufkirchan; 793590), 1.2 KH_2_PO_4_ (Sigma‐Aldrich Chemie GmbH, Taufkirchan; P0662), 11.1 glucose (Sigma‐Aldrich Chemie GmbH, Taufkirchan; RDD016), 1.5 CaCl_2_ (Sigma‐Aldrich Chemie GmbH, Taufkirchan; 21115), 2 Na‐pyruvate (Sigma‐Aldrich Chemie GmbH, Taufkirchan; P5280) and bubbled with 95% O_2_ and 5% CO_2_ at 37°C). Perfusion pressure was maintained at 70 mm Hg. Ex vivo ECGs in isolated heart were recorded from the outer side of the explanted heart. Negative and positive ends were placed at the root of the aorta and apex of the ventricles, respectively. Endocardial mapping of the “right heart” (right atrium and right ventricle) was done at the inner side of the explanted heart using octopolar silver electrodes. The closest derivation to the atrioventricular node was used for the electrical activation of the right atrium (A wave) (“Ch5” in Figure 6a) and the derivation at the apex of the right ventricle for the electrical activation of the right ventricle (V wave) (“Ch1” in Figure 6a). Power lab bio‐amplifiers collected the ECG tracings, which were low‐ and high‐pass filtered (at 5 kHz and 30 Hz, respectively) and sampled at a speed of 40 k/s. Initially, the heart was perfused with KHB for the first 10 min, after which a solution of 5 μM NBA, 5 μM CBA or vehicle control (DMSO 0.05%) dissolved in KHB was added to the perfusion. Data were analyzed using Lab Chart Pro V8. (AD. Instruments, Australia). Only the heart rate is presented due to the lack of accuracy in the other parameters. Visualization of at least six endocardial ECG leads at the level of the right atrium and right ventricle establishes the correct position of the catheter. Atrioventricular delay was measured between channels 5 and 6 as it displayed waves A and V at the Ch1. The hearts beat according to their sinus rhythm and were not paced externally during the procedure.

### 2.7 RNA preparation and real‐time quantitative RT‐PCR

Isolated RNA from atrial and ventricular myocytes was extracted and amplified using PCR. Both left and right atrial myocytes were used from three mice for each genotype. TaqMan gene expression assay probes for mouse *Trpm4* (Mm01205532‐m1) and *Gapdh* (Mm99999915‐g1) were used to amplify the isolated RNA. Comparative threshold (CT) values and target *Trpm4* genes were determined for reference for each sample set. Relative quantification was then performed using the CT method (ΔΔCT).

### 2.8 Cell line preparations, transfection, and whole‐cell electrophysiology

Human embryonic kidney cells (HEK‐293) were cultured in DMEM (Gibco, Basel, Switzerland; 41965) supplemented with 10% FBS, 0.5% penicillin, and streptomycin (10,000 U/mL, Invitogene, Switzerland; 15140‐122) at 37°C in a 5% CO_2_ incubator. For electrophysiological studies, T25 flasks with HEK‐293 cells were transiently cotransfected using X‐tremeGENE™ 9 reagent (Sigma‐Aldrich, Darmstadt, Germany; 6365779001) with 2 μg of mouse Na_v_1.5 cDNA coding for the α‐subunit (without β‐subunits). All transfections included 0.1 μg of cDNA encoding the GFP protein. Forty‐eight hours post‐transfection, green cells were selected and analyzed for patch clamp experiments. Peak sodium currents (*I*_Na_) were recorded in the whole‐cell configuration at RT (22–23°C) using a VE‐2 amplifier (Alembic Instrument, USA). Sodium currents were digitized at a sampling frequency of 20 kHz. Borosilicate glass pipettes were pulled to a series resistance of ~2 MΩ. pClamp software, version 8 (Axon Instruments, Union City, CA, USA) was used for recordings and data analysis. HEK‐293 cells were bathed in a solution containing (in mM) NaCl 30, NMDG‐Cl 100 (Sigma‐Aldrich Chemie GmbH, Taufkirchan; 66930), CaCl_2_ 2, MgCl_2_ 1.2, CsCl 5 (Sigma‐Aldrich Chemie GmbH, Taufkirchan; 289329), HEPES 10, and glucose 5 (pH was adjusted to 7.4 with CsOH (HuberLab, Aesch, Switzerland; 21000)). HEK‐293 cells were initially voltage‐clamped (holding potential −80 mV) and dialyzed with an internal solution containing (in mM) CsCl 60, Cs‐aspartate 70 (Sigma‐Aldrich Chemie GmbH, Taufkirchan; A91310‐0), CaCl_2_ 1, MgCl_2_ 1, HEPES 10, EGTA 11, and Na_2_ATP 5 (Sigma‐Aldrich Chemie GmbH, Taufkirchan; A26209) (pH was adjusted to 7.2 with CsOH). A pulse protocol consisting to a depolarization step for 20 ms from −80 mV to −20 mV was applied every 3 s. The sodium peak current was first recorded under vehicle perfusion (DMSO 0.05% (Sigma‐Aldrich Chemie GmbH, Taufkirchan; 41,648)) for 1 min (or until stability was reached). Then, the compound (NBA or CBA 5 μM) or vehicle (DMSO 0.05%) was applied for 5 min (or until recordings had stabilized). The sodium peak currents measured during the stable phase during the vehicle, and compound/vehicle perfusion were used to quantify the percentage of the effect of each compound or the vehicle using the following formula:

Percentage of effect on sodium current = 100‐(100*I_Na compounds/vehicle_/*I*_Na vehicle_).

### 2.9 Drugs

Stock solutions of 4‐chloro‐2‐(2‐(naphthalene‐1‐ yloxy) acetamido) benzoic acid (NBA) and 4‐chloro‐ 2‐[2‐(2‐chloro‐phenoxy)‐acetylamino]‐benzoic acid (CBA) were made in 100% DMSO to a final concentration of 10 mM. Before each experiment, fresh NBA and CBA solutions at 5 μM (DMSO 0.05%) were made using either Krebs–Henseleit buffer (endocardial mapping) or extracellular solution (patch clamp). The final concentration of NBA was chosen based on the previously determined concentration estimated to fully block mouse TRPM4 channel overexpressed in a heterologous expression system (Arullampalam et al., 2021). Given that CBA failed to inhibit mouse TRPM4 in a heterologous expression system, this compound was chosen as a negative control for experiments (Arullampalam et al., 2021).

### 2.10 Data analyses and statistics

Data are represented as means ± SEM. Statistical analyses were performed using Prism7 GraphPad™ software (GraphPad by Dotmatics, San Diego, CA, USA). An unpaired nonparametric *t*‐test followed by a Mann–Whitney U posttest was used to compare two unpaired groups. A paired nonparametric *t*‐test followed by a Wilcoxon matched‐pairs signed‐rank posttest was used to compare two paired groups. *p* < 0.05 was considered significant. No muti‐group comparison has been performed in this study. No data were deidentified prior analysis and no randomization design has been used for the experiments. The different measurements between groups were done at similar times of the day (between 10:00 am and 4:00 pm).

## 3 RESULTS

### 3.1 TRPM4 expression is downregulated in *Trpm4* ^−/−^ mouse heart

**FIGURE 1:**
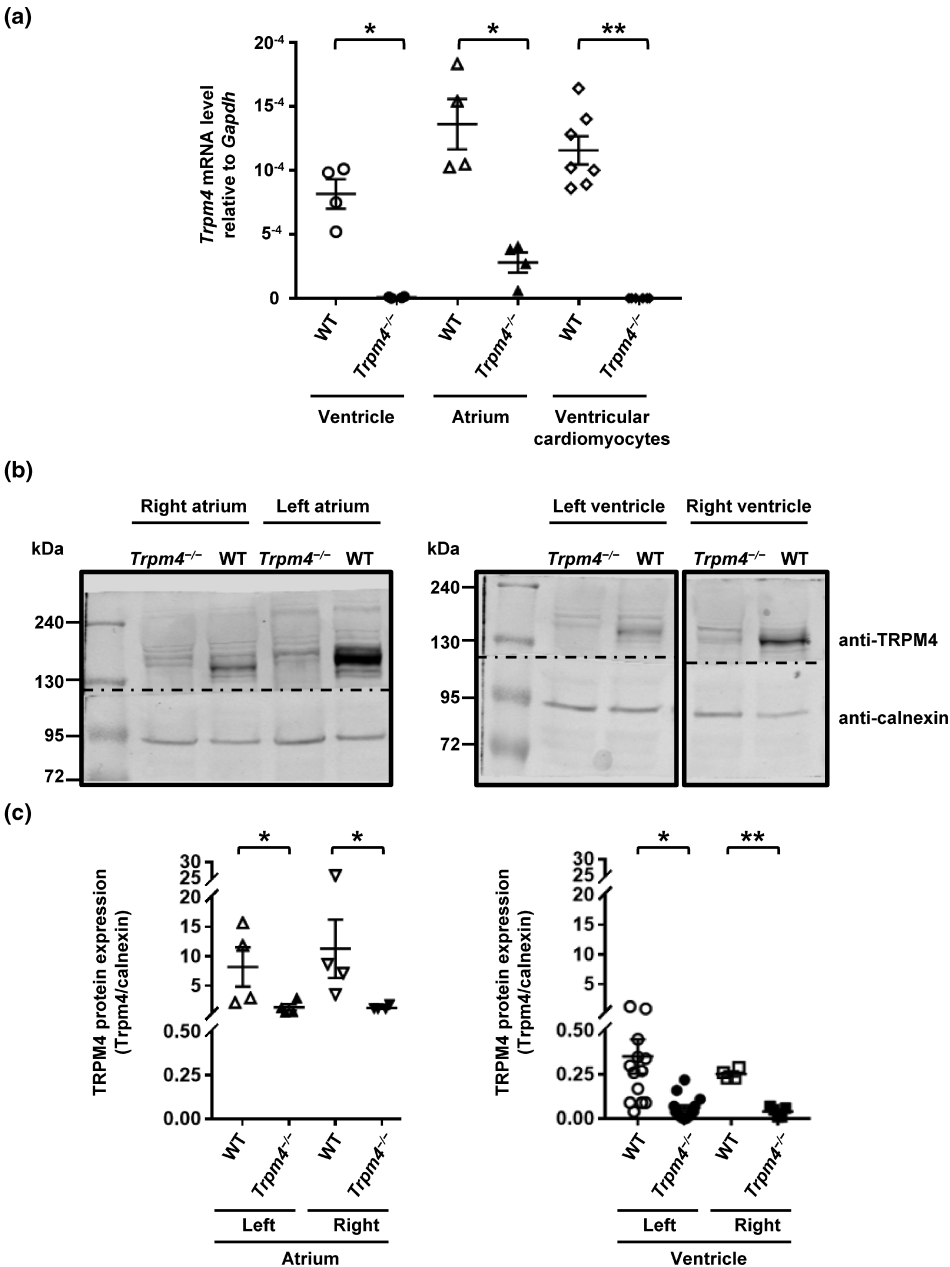
Trpm4 expression in mouse heart. (a) Quantification of *Trpm4* mRNA using RT‐qPCR. For the ventricle, atrium, and ventricular cardiomyocytes, Ct values were corrected with the reference gene *Gapdh*. *p ⩽ 0.05 and **p ⩽ 0.01 (*n* ⩾ 4 per group). (b) Expression of TRPM4 in wild‐type and *Trpm4* ^−/−^ mouse atrial and ventricular tissues. Representative immunoblots of TRPM4 protein expression from the right atrium, left atrium, right ventricle, and left ventricle. Dotted lines indicate cut in Western blot membranes. (c) Quantification of the immunoblots showing protein expression from the right atrium, left atrium, right ventricle, and left ventricle (Y‐axis is split for clarity). * *p* ⩽ 0.05 and **p ⩽ 0.01 (*n* ⩾ 4 per group). An unpaired nonparametric *t*‐test followed by a Mann–Whitney U posttest was used to compare two unpaired groups (panels a and c).

The role of TRPM4 in cardiac function has been investigated with a new *Trpm4* ^−/−^ mouse model, in which exon 10 of the *Trpm4* gene has been deleted (Figure S1) (Ozhathil et al., 2021). The *Trpm4* ^−/−^ mouse model investigated here did not show any increase in mortality or alteration of Mendelian genetic transmission. At adult age, body weight was 9.0% lower in adult male *Trpm4* ^−/−^ only compared to their controls ((average body weight _wild‐type adult male_: 33.21 ± 0.62 gram (*n* = 28); average body weight _*Trpm4*−/− adult male_: 30.21 ± 0.60 gram (*n* = 26); *p* < 0.01) and (average body weight _wild‐type adult female_: 25.43 ± 0.99 gram (*n* = 25); average body weight _*Trpm4*−/− adult female_: 24.03 ± 0.45 gram (*n* = 24); ns)) (Figure S2). As expected, cardiac TRPM4 expression in *Trpm4* ^−/−^ hearts is drastically decreased in the heart compared to wild‐types, both at the mRNA level (Figure 1a) and at the protein level (Figure 1b,c) as previously reported (Ozhathil et al., 2021). In addition, we investigated the expression level of TRPM4 protein in the four chambers (left and right atria and left and right ventricles) of wild‐type adult mouse heart. Quantification of Western blots shown no significant difference either between the left and right atrium or between le left and right ventricle (Figure 1c). However, TRPM4 protein seems to be comparatively more expressed in atria than in ventricles which may reflect its predominant role in atrial level (normalized TRPM4 protein expression in atrium: 9.7 ± 2.8 (*n* = 8) and normalized TRPM4 protein expression in ventricles: 0.3 ± 0.1 (*n* = 14); *p* < 0.001) (Figure 1c).

### 3.2 *Trpm4* knockdown only affects surface electrocardiograms parameters in male compared to controls

**FIGURE 2:**
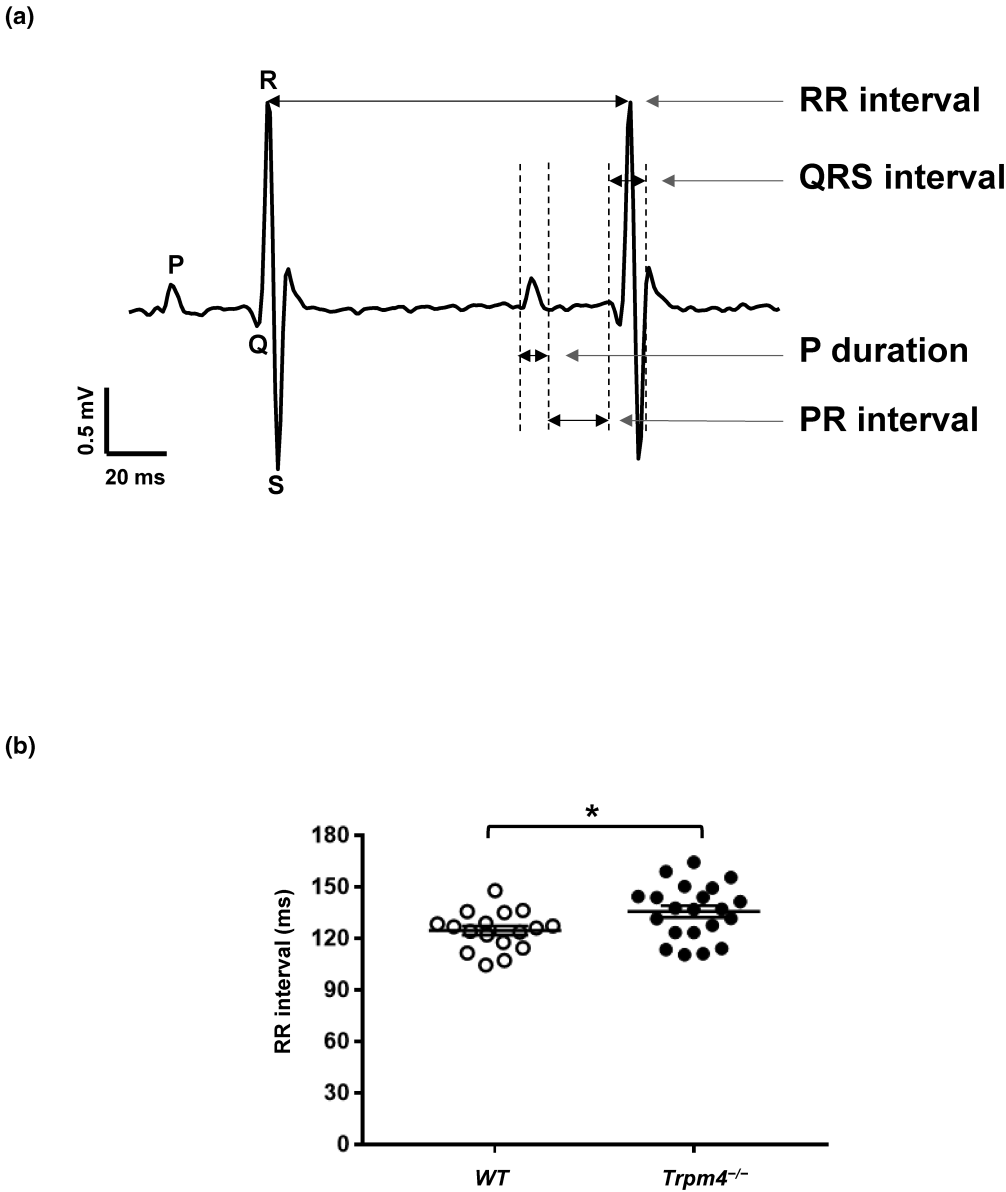
ECG parameters in wild‐type and *Trpm4* ^−/−^ mice. (a) Representative ECG trace from adult male wild‐type mouse and the different parameters investigated. (b) RR interval showing the difference between male 24‐week‐old wild‐type and *Trpm4* ^−/−^ mice. *p ⩽ 0.05 (*n* ⩾ 13 per group). An unpaired nonparametric *t*‐test followed by a Mann–Whitney U posttest was used to compare two unpaired groups.

Surface ECGs (Figure 2a) were recorded to assess the cardiac electrophysiology of eight groups of mice (young male and female wild‐type, young male and female *Trpm4* ^−/−^, adult male and female wild‐type, and adult male and female *Trpm4* ^−/−^). Before these ECGs were performed, cardiac electrical parameters were first characterized in male and female *Trpm4* ^−/−^ and wild‐type mice by recording weekly ECGs in 5‐ to 9‐week‐old animals (Table 1). These ECGs did not reveal any differences in the measured parameters (RR interval, PR interval, QRS interval, and P duration) between the different groups (Table 1). Moreover, most of the investigated ECG parameters did not differ between young and adult mice of both sexes and with wild‐type and *Trpm4* ^−/−^ genetic backgrounds (Table 1). Only a significant HR decreases of 8% ± 2%, with a concomitant increase of the RR interval, was observed in young and adult male *Trpm4* ^−/−^ mice compared to male wild‐type animals (Figure 2b, and Table 1). Quantifying the HRV using the RMSSD index did not showed any alteration between wild‐type and *Trpm4* ^−/−^ animals neither in male nor in female (Table 1).

**TABLE 1:** Evolution of the ECG parameters in mice at 5‐, 6‐, 7‐, 8‐, and 9‐weeks of age, at a young age (12‐week‐old) and adult age (more than 24‐week‐old).

		5th week	6th week	7th week	8th week	9th week	Young	Adult
RR interval (ms)								
Wild‐type	♂	136.5 ± 3.0 (*n* = 4)	119.2 ± 4.8 (*n* = 4)	128.7 ± 2.5 (*n* = 4)	124.5 ± 0.9 (*n* = 4)	124.6 ± 2.2 (*n* = 4)	122.6 ± 2.4 (*n* = 16)	124.5 ± 2.7 (*n* = 17)
*Trpm4* ^−/−^		135.8 ± 3.8 (*n* = 5)	127.6 ± 5.1 (*n* = 5)	117.7 ± 3.5 (*n* = 5)	127.6 ± 5.4 (*n* = 5)	120.4 ± 2.9 (*n* = 5)	131.6 ± 3.6 (*n* = 13)	135.7 ± 3.5 (*n* = 21)
Wild‐type	♀	144.8 ± 3.2 (*n* = 4)	121.5 ± 5.5 (*n* = 4)	124.2 ± 2.7 ( *n* = 4)	117.9 ± 4.8 (*n* = 4)	107.3 ± 1.8 (*n* = 4)	127.1 ± 2.8 (*n* = 11)	137.6 ± 3.2 (*n* = 7)
*Trpm4* ^−/−^		151.3 ± 12.6 (*n* = 3)	145.5 ± 2.4 (*n* = 3)	133.8 ± 12.0 (*n* = 3)	131.5 ± 2.0 (*n* = 3)	134.1 ± 5.6 (*n* = 3)	133.4 ± 7.7 (*n* = 8)	143.8 ± 3.5 (*n* = 9)
PR interval (ms)								
Wild‐type	♂	34.8 ± 1.0 (*n* = 4)	31.7 ± 1.7 (*n* = 4)	35.0 ± 1.8 (*n* = 4)	35.8 ± 1.5 (*n* = 4)	35.9 ± 1.4 (*n* = 4)	39.5 ± 0.6 (*n* = 16)	40.5 ± 0.7 (*n* = 17)
*Trpm4* ^−/−^		37.6 ± 1.2 (*n* = 5)	38.1 ± 0.6 (*n* = 5)	37.2 ± 0.8 (*n* = 5)	38.0 ± 0.7 (*n* = 5)	36.6 ± 0.8 (*n* = 5)	40.4 ± 0.7 (*n* = 13)	41.2 ± 0.7 (*n* = 21)
Wild‐type	♀	37.5 ± 1.1 (*n* = 4)	36.5 ± 0.6 (*n* = 4)	36.6 ± 0.6 (*n* = 4)	37.8 ± 2.7 (*n* = 4)	39.6 ± 1.6 (*n* = 4)	40.3 ± 1.2 (*n* = 11)	41.8 ± 1.0 (*n* = 7)
*Trpm4* ^−/−^		35.9 ± 1.8 (*n* = 3)	38.1 ± 1.3 (*n* = 3)	36.2 ± 0.8 (*n* = 3)	38.6 ± 1.7 (*n* = 3)	35.9 ± 0.4 (*n* = 3)	40.2 ± 1.7 (*n* = 8)	42.3 ± 1.0 (*n* = 9)
QRS interval (ms)								
Wild‐type	♂	10.5 ± 0.8 (*n* = 4)	9.8 ± 0.5 (*n* = 4)	11.2 ± 0.5 (*n* = 4)	10.7 ± 0.5 (*n* = 4)	11.2 ± 0.0 (*n* = 4)	10.4 ± 0.4 (*n* = 16)	10.1 ± 0.3 (*n* = 17)
*Trpm4* ^−/−^		10.5 ± 0.5 (*n* = 5)	10.3 ± 0.6 (*n* = 5)	10.4 ± 0.6 (*n* = 5)	10.5 ± 0.5 (*n* = 5)	11.1 ± 0.3 (*n* = 5)	9.8 ± 0.4 (*n* = 13)	10.9 ± 0.3 (*n* = 21)
Wild‐type	♀	10.7 ± 0.6 (*n* = 4)	10.6 ± 0.4 (*n* = 4)	10.9 ± 0.2 (*n* = 4)	10.4 ± 0.5 (*n* = 4)	10.8 ± 0.5 (*n* = 4)	10.9 ± 0.3 (*n* = 11)	11.0 ± 0.5 (*n* = 7)
*Trpm4* ^−/−^		11.8 ± 0.6 (*n* = 3)	11.2 ± 0.8 (*n* = 3)	11.3 ± 0.1 (*n* = 3)	12.3 ± 0.3 (*n* = 3)	11.4 ± 0.5 (*n* = 3)	11.2 ± 0.5 (*n* = 8)	10.2 ± 0.5 (*n* = 9)
P duration (ms)								
Wild‐type	♂	8.8 ± 0.5 (*n* = 4)	8.4 ± 0.4 (*n* = 4)	10.4 ± 0.4 (*n* = 4)	9.8 ± 0.3 (*n* = 4)	10.0 ± 0.5 (*n* = 4)	16.7 ± 1.2 (*n* = 16)	14.4 ± 1.0 (*n* = 17)
*Trpm4* ^−/−^		11.0 ± 0.7 (*n* = 5)	11.0 ± 0.8 (*n* = 5)	10.4 ± 0.4 (*n* = 5)	11.1 ± 0.4 (*n* = 5)	10.7 ± 0.6 (*n* = 5)	15.0 ± 1.2 (*n* = 13)	17.1 ± 1.3 (*n* = 21)
Wild‐type	♀	10.4 ± 0.3 (*n* = 4)	9.3 ± 0.5 (*n* = 4)	9.8 ± 0.5 (*n* = 4)	10.0 ± 1.2 (*n* = 4)	8.4 ± 0.8 (*n* = 4)	13.5 ± 1.5 (*n* = 11)	19.0 ± 1.0 (*n* = 7)
*Trpm4* ^−/−^		10.9 ± 0.3 (*n* = 3)	11.1 ± 0.9 (*n* = 3)	11.1 ± 0.6 (*n* = 3)	11.9 ± 0.6 (*n* = 3)	10.3 ± 0.7 (*n* = 3)	16.3 ± 2.0 (*n* = 8)	15.3 ± 1.7 (*n* = 9)
HRV‐RMSSD (ms)								
Wild‐type	♂	n.d	n.d	n.d	n.d	n.d	2.331 ± 0.362 (*n* = 6)	1.820 ± 0.183 (*n* = 13)
*Trpm4* ^−/−^		n.d	n.d	n.d	n.d	n.d	2.298 ± 0.287 (*n* = 10)	2.375 ± 0.255 (*n* = 12)
Wild‐type	♀	n.d	n.d	n.d	n.d	n.d	2.614 ± 0.764 (*n* = 8)	3.008 ± 0.960 (*n* = 4)
*Trpm4* ^−/−^		n.d	n.d	n.d	n.d	n.d	2.031 ± 0.464 (*n* = 6)	3.019 ± 0.718 (*n* = 8)
HR (bpm)								
Wild‐type	♂	440 ± 10 (*n* = 4)	506 ± 19 (*n* = 4)	467 ± 9 (*n* = 4)	482 ± 4 (*n* = 4)	482 ± 8 (*n* = 4)	492 ± 9 (*n* = 16)	486 ± 11 (*n* = 17)
*Trpm4* ^−/−^		443 ± 12 (*n* = 5)	443 ± 19 (*n* = 5)	512 ± 15 (*n* = 5)	474 ± 20 (*n* = 5)	499 ± 12 (*n* = 5)	460 ± 13 (*n* = 13)	448 ± 12 (*n* = 21)
Wild‐type	♀	415 ± 9 (*n* = 4)	497 ± 22 (*n* = 4)	484 ± 11 (*n* = 4)	512 ± 23 (*n* = 4)	560 ± 10 (*n* = 4)	474 ± 11 (*n* = 11)	437 ± 10 (*n* = 7)
*Trpm4* ^−/−^		402 ± 31 (*n* = 3)	413 ± 7 (*n* = 3)	456 ± 40 (*n* = 3)	457 ± 7 (*n* = 3)	449 ± 19 (*n* = 3)	461 ± 28 (*n* = 8)	420 ± 10 (*n* = 9)

Abbreviation: n.d., not determined.

*Note*: ECG parameters from male and female wild‐type and *Trpm4* ^*−/−*^ mice at different ages are shown as indicated. Values highlighted in light gray (*p ⩽ 0.05) indicate a statistical difference between *Trpm4* ^−/−^ and wild‐type for the respective matching age and sex group. bpm, beats per minute.

### 3.3 Echocardiography investigations shown cardiac hypertrophy in adult male *Trpm4* ^−/−^ mice

**FIGURE 3:**
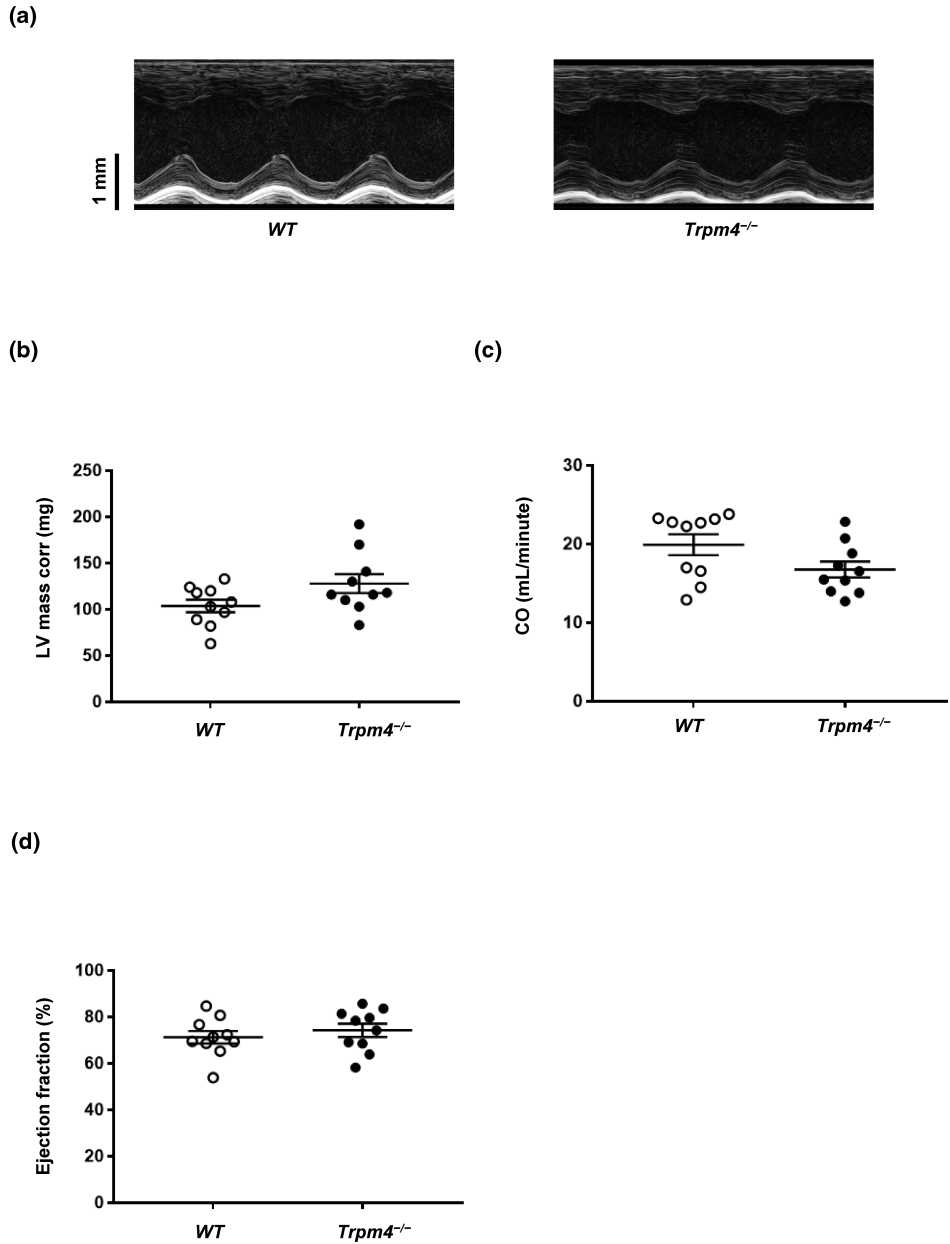
Left ventricle structure and function of wild‐type and *Trpm4* ^−/−^ mouse hearts. (a) Raw captions from wild‐type and *Trpm4* ^−/−^ mouse hearts from the parasternal, short axis, M‐mode echocardiography. (b–d) left ventricle mass corrected (LV Mass corr), cardiac output (CO), and ejection fraction parameters recorded in adult male wild‐type and *Trpm4* ^−/−^ mice suggesting an increase of the left ventricle mass of *Trpm4* ^−/−^ mice (*b*) and a decrease of the cardiac output (*c*) without systolic dysfunction (*d*) (*n* = 10 per group). An unpaired nonparametric *t*‐test followed by a Mann–Whitney U posttest was used to compare two unpaired groups (panels b–d).

**FIGURE 4:**
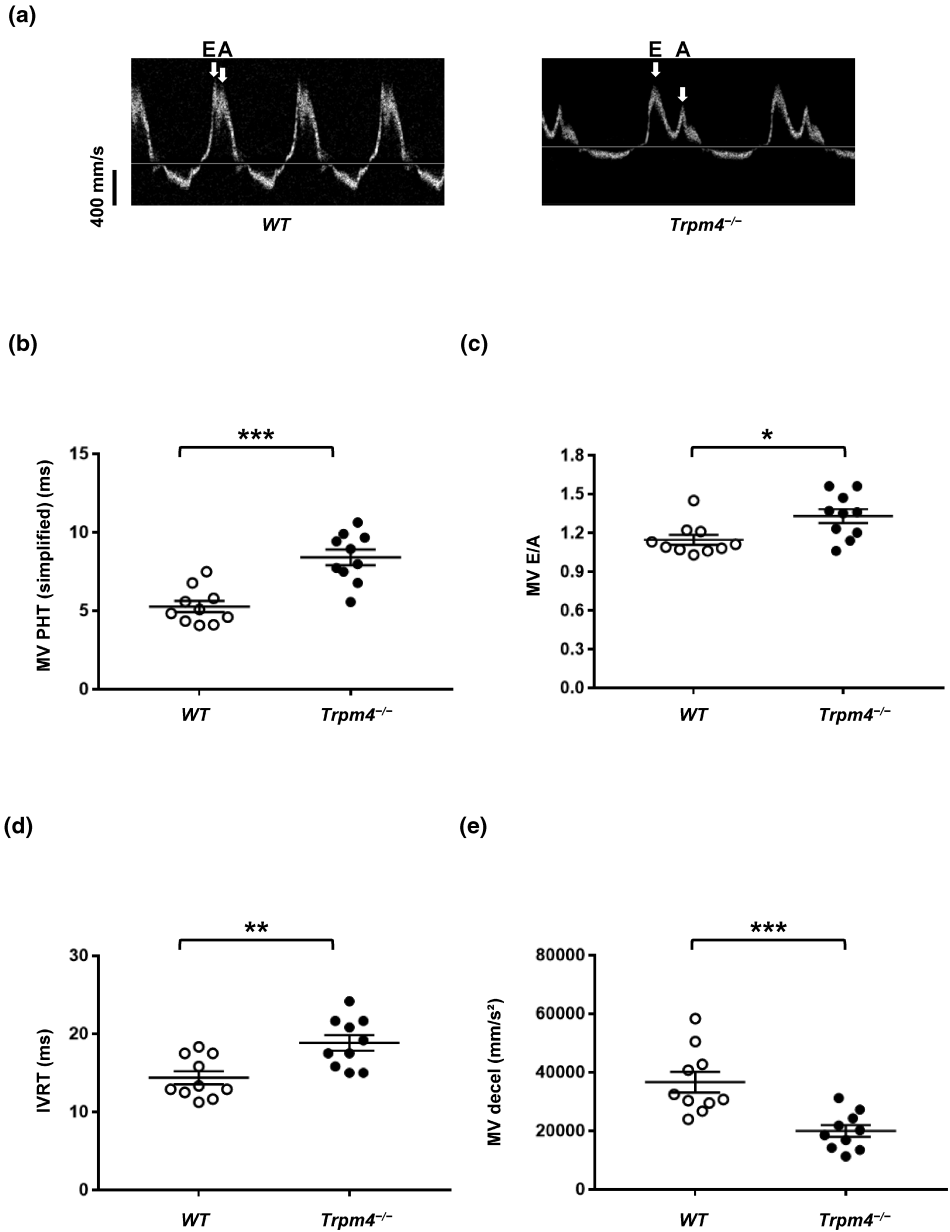
Analysis of mitral valve function of wild‐type and *Trpm4* ^−/−^ mouse hearts. (a) Traces representing the blood flow velocity, with the E and A waves, through the mitral valves of male wild‐type (left) and *Trpm4* ^−/−^ (right) hearts. (b–e) Dot plots showing the mitral valve pressure halftime (MV PHT) (*b*), the mitral valve E/A ratio (MV E/A), which correspond to the ratio between the velocities of E wave (early filling) and A wave (atrial filling) of the mitral valve (*c*), the mitral valve isovolumetric relaxation time (IVRT) (*d*), and the mitral valve deceleration time MV (decel) (*e*). *p ⩽ 0.05, **p ⩽ 0.01, and ***p ⩽ 0.001 (*n* = 10 per group). An unpaired nonparametric t‐test followed by a Mann–Whitney U posttest was used to compare two unpaired groups (panels b–e).

**FIGURE 5:**
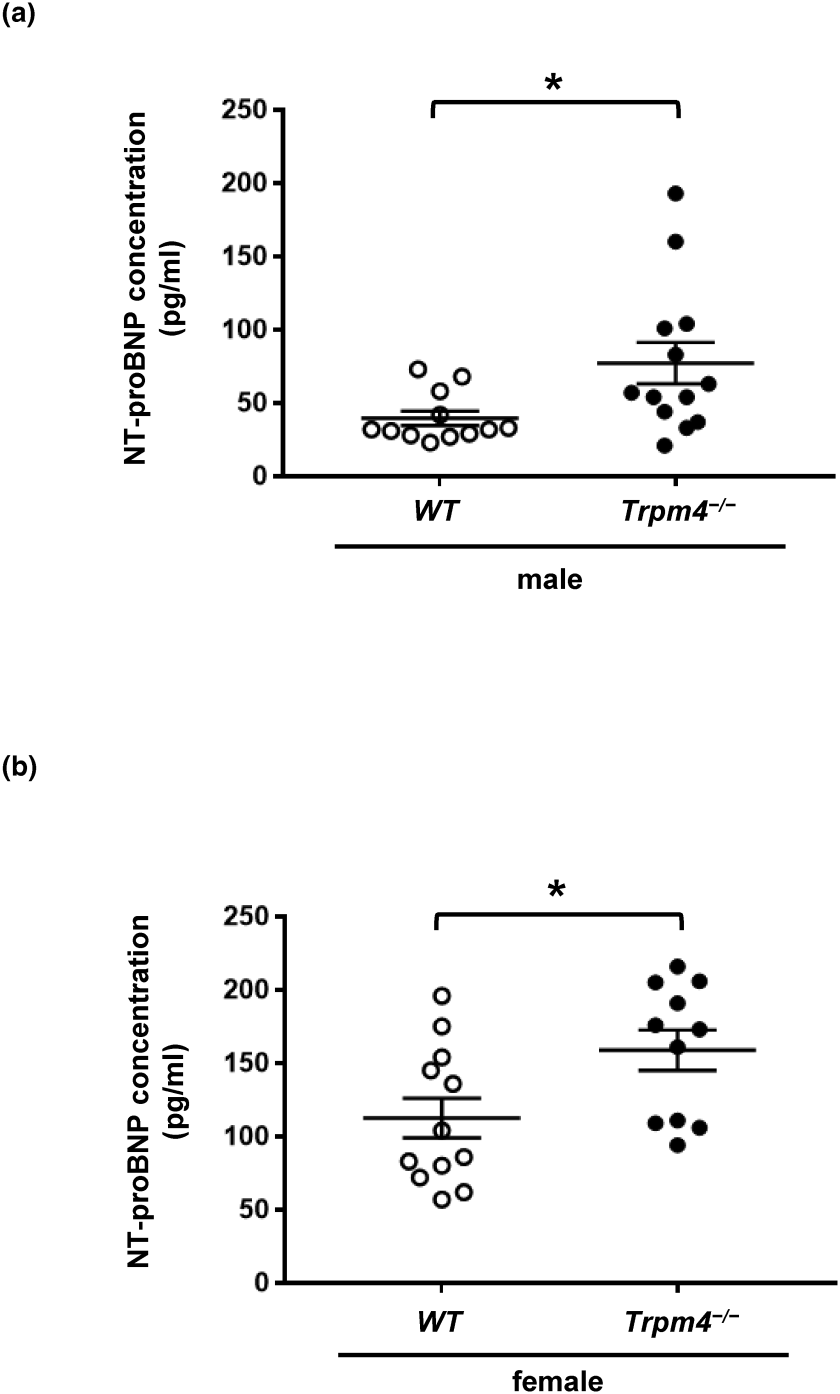
N‐terminal pro‐brain natriuretic peptide concentration is increase in adult *Trpm4* ^−/−^ sera. (a) Dot blots showing the NT‐proBNP concentration in adult male *Trpm4* ^−/−^ sera. (b) Dot blots showing the NT‐proBNP concentration in adult female *Trpm4* ^−/−^ sera. *p ⩽ 0.05 (*n* ⩾ 11 per group). An unpaired nonparametric *t*‐test followed by a Mann–Whitney U posttest was used to compare two unpaired groups (panels a and b).

To characterize the left ventricle, we studied the following diastolic and systolic functions of *Trpm4* ^−/−^ and wild‐type mice using echocardiography: stroke volume, ejection fraction, fractional shortening, cardiac output, left ventricle mass corrected, mitral valve pressure halftime simplified, mitral valve deceleration time, left ventricle isovolumetric relaxation time, and mitral valve E/A ratio (Tables 2 and 3). The systolic function of the left ventricle was evaluated using the parasternal short‐axis view of the M mode (Figure 3a). The diastolic function was assessed using power Doppler imaging to obtain an apical four‐chamber view (Figures 4a and 5a). Scans of the hearts of young animals revealed that the mitral valve pressure halftime (MV‐PHT) and the left ventricle isovolumetric relaxation time (IVRT) were significantly increased in *Trpm4* ^−/−^ male animals compared to wild‐type controls (Table 3). Other parameters did not differ between wild‐type and *Trpm4* ^−/−^ animals for both sexes (Tables 2 and 3). At adult age, however, *Trpm4* ^−/−^ males presented with a higher left ventricular mass (corrected values) than wild‐types, which is coherent with a slightly reduction in cardiac output compared to their respective controls (Figure 3b, c, and Table 2). The ejection fraction, which reflects the systolic function, was unaltered in male adult *Trpm4* ^−/−^ animals compared to their controls (Figure 3d and Table 2). Conversely, the left ventricle mass corrected values of *Trpm4* ^−/−^ females tended to be smaller than their controls (Table 2). These results suggest that male adult *Trpm4* ^−/−^ animals tend to develop left ventricle hypertrophy with age without systolic dysfunction. Interestingly, the remaining average mitral valve pressure (halftime simplified) was increased in male adult *Trpm4* ^−/−^ mice compared to the wild‐type animals (Figure 4b, and Table 3), suggesting a mitral valve dysfunction. Therefore, we recorded other mitral valve function parameters (mitral valve E/A ratio, left ventricle isovolumetric relaxation time, and mitral valve deceleration time), and found, they were all significantly altered in adult male *Trpm4* ^−/−^ animals compared to wild‐type (Figure 4c–e, and Table 3). Female adult *Trpm4* ^−/−^ mice, compared to their controls, also showed alteration of different parameters related to the mitral valve function (Table 3).

**TABLE 2:** Echocardiography parameters related to the left ventricular structure and function. Left ventricle parameters from young and adult, male and female, and wild‐type and *Trpm4* ^*−/−*^ mice.

		Stroke volume (μl)	Ejection fraction (%)	Fractional shortening (%)	Cardiac output (mL/minute)	LV mass corrected (mg)
Young						
Wild‐type	♂	39.4 ± 5.5 (*n* = 5)	71.8 ± 2.1 (*n* = 5)	40.3 ± 1.8 (*n* = 5)	20.601 ± 2.934 (*n* = 5)	94 ± 8 (*n* = 5)
*Trpm4* ^−/−^		36.8 ± 1.4 (*n* = 5)	72.1 ± 4.4 (*n* = 5)	41.1 ± 3.7 (*n* = 5)	16.297 ± 0.821 (*n* = 5)	86 ± 6 (*n* = 5)
Wild‐type	♀	35.7 ± 3.2 (*n* = 4)	74.4 ± 2.1 (*n* = 4)	42.4 ± 1.8 (*n* = 4)	14.647 ± 1.338 (*n* = 4)	66 ± 2 (*n* = 4)
*Trpm4* ^−/−^		38.8 ± 2.9 (*n* = 4)	76.5 ± 3.9 (*n* = 4)	44.8 ± 3.8 (*n* = 4)	16.500 ± 0.725 (*n* = 4)	87 ± 13 (*n* = 4)
Adult						
Wild‐type	♂	43.4 ± 1.6 (*n* = 10)	71.3 ± 2.7 (*n* = 10)	40.4 ± 2.2 (*n* = 10)	19.909 ± 1.320 (*n* = 10)	104 ± 7 (*n* = 10)
*Trpm4* ^−/−^		39.3 ± 1.9 (*n* = 10)	74.3 ± 2.9 (*n* = 10)	43.0 ± 2.4 (*n* = 10)	16.754 ± 1.021 (*n* = 10)	128 ± 10 (*n* = 10)
Wild‐type	♀	41.3 ± 2.8 (*n* = 8)	69.6 ± 2.0 (*n* = 8)	38.7 ± 1.6 (*n* = 8)	17.930 ± 1.185 (*n* = 8)	93 ± 7 (*n* = 8)
*Trpm4* ^−/−^		37.3 ± 3.1 (*n* = 10)	73.3 ± 2.1 (*n* = 10)	41.7 ± 1.8 (*n* = 10)	15.187 ± 1.396 (*n* = 10)	73 ± 8 (*n* = 10)

**TABLE 3:** Echocardiography parameters related to the mitral valve structure and function. Mitral valve parameters from young and adult, male and female, and wild‐type and *Trpm4* ^−*/*−^ mice.

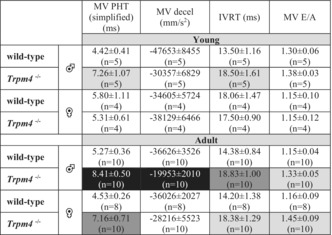

Abbreviations: IVRT, left ventricle isovolumetric relaxation time; MV decel, mitral valve deceleration time; MV E/A, mitral valve E/A ratio; MV PHT: mitral valve pressure half time.

*Note*: Values highlighted in light grey (*p⩽0.05), dark gray (**p⩽0.01), and black (***p⩽0.001) indicate a statistical difference between *Trpm4* ^−*/*−^ and wild‐type for the respective matching age and sex group.

To further investigate the left ventricular hypertrophy observed using echocardiography, N‐terminal pro‐brain natriuretic peptide (NT‐proBNP) concentration was measured in sera from adult male and female mice. NT‐proBNP is a cardiac biomarker that indicates myocyte stretch and is commonly used in the clinical setting to assess left ventricular wall stress, especially in the context of heart failure (Welsh et al., 2022). During pressure or volume overload, cardiomyocytes secrete the prohormone proBNP, which is then cleaved into the diuretic and vasodilative BNP and the biologically inactive by‐product NT‐proBNP. Therefore, NT‐pro BNP is significantly elevated in conditions such as ventricular hypertrophy, valvular heart disease, atrial fibrillation, and pulmonary hypertension. In analogy to the findings from Raymond et al. who showed that women had higher NT‐proBNP levels than men, we show that female mice had higher NT‐proBNP concentrations than male mice. This observation was significant both in wild‐type and *Trpm4* ^−/−^ mice. (Figure 5a,b) (Raymond et al., 2003). Moreover, the NT‐proBNP concentration was increased in male and female *Trpm4* ^−/−^ compared to wild‐type sera suggesting that the knockdown of TRPM4 protein leads to increased ventricular wall stress (Figure 5a,b). However, the relative increase in NT‐proBNP was stronger in male than female mice ((NT‐proBNP _wild‐type adult male_: 100 ± 12% (*n* = 12); NT‐proBNP _*Trpm4*−/− adult male_: 195 ± 36% (*n* = 13); *p* < 0.05) and (NT‐proBNP _wild‐type adult female_: 100 ± 12% (*n* = 12); NT‐proBNP _*Trpm4*−/− adult female_: 141 ± 12% (*n* = 11); *p* < 0.05)). This is in line with our echocardiography data suggesting that, compared to their controls, male *Trpm4* ^−/−^ animals tend to develop left ventricle hypertrophy when they age, while female *Trpm4* ^−/−^ mice do not. However, male and female *Trpm4* ^−/−^ mice present similar mitral valve dysfunctions.

### 3.4 *Trpm4* knockdown does not affect right ventricle echocardiography parameters

**FIGURE 6:**
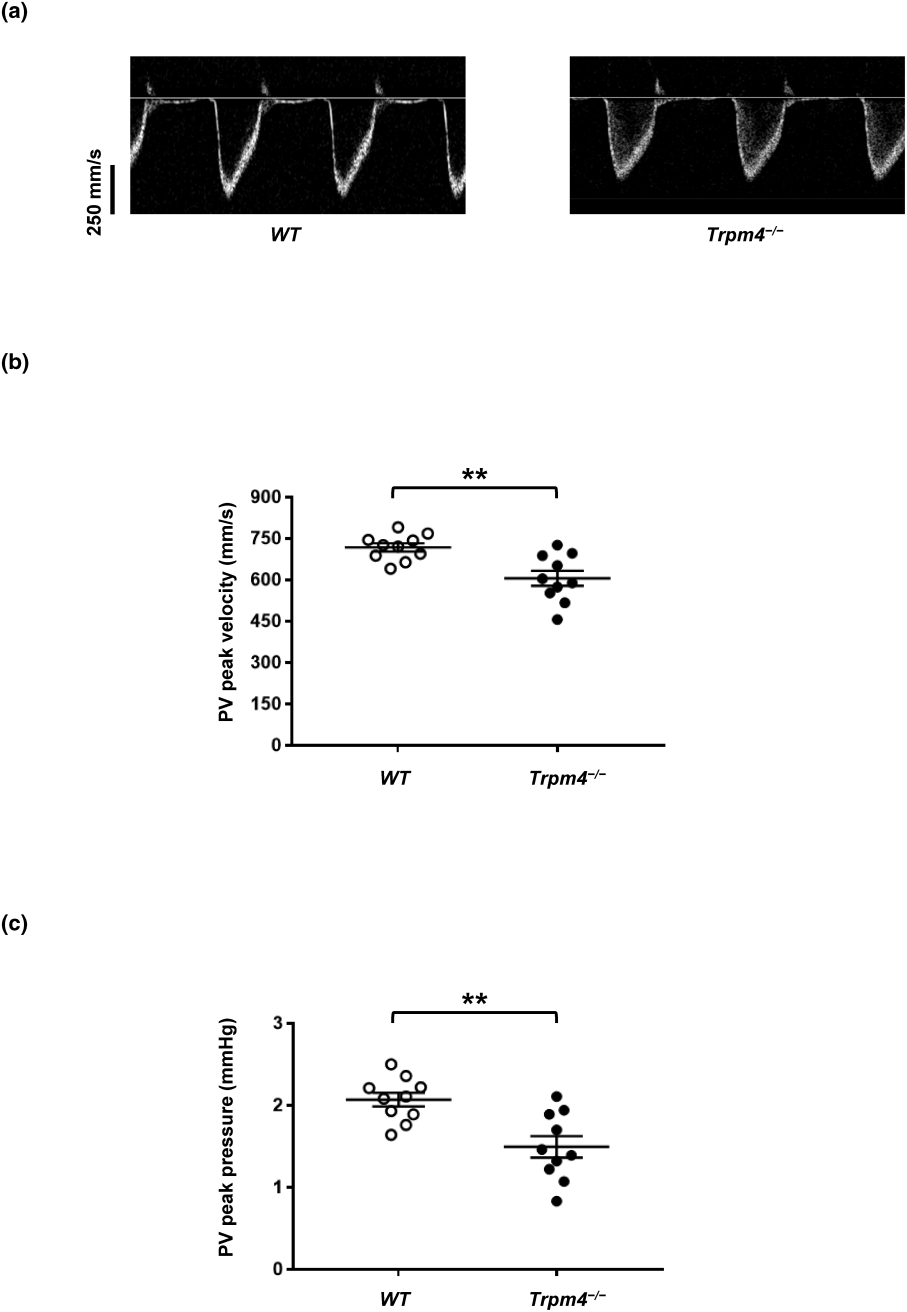
Analysis of pulmonary artery blood flow of wild‐type and *Trpm4* ^−/−^ mouse hearts. (a) Traces representing the velocity of blood flow in the pulmonary artery of male wild‐type (left) and *Trpm4* ^−/−^ (right) hearts. (b,c) Dot blots showing the pulmonary valve peak velocity (b) and the pulmonary valve peak pressure (c). **p ⩽ 0.01 (*n* = 10 per group). An unpaired nonparametric *t*‐test followed by a Mann–Whitney U posttest was used to compare two unpaired groups (panels b and c).

Visualizing the right ventricle with power Doppler imaging proved technically challenging as the sternum obstructed the view; therefore, we could not obtain an apical four‐chamber view. Instead, we indirectly investigated the right ventricle systolic function by scanning the pulmonary artery and valve, determining pulmonary valve peak velocity, pulmonary valve diameter, pulmonary valve peak pressure, and the mean pulmonary internal pressure (mPAP common). In young animals of both sexes, no alterations in the different parameters were observed (Table 4). In male adult animals, only the pulmonary valve peak velocity and the pulmonary valve peak pressure were significantly lower in male *Trpm4* ^−/−^ animals compared to their controls suggesting an alteration of the right ventricle of adult male *Trpm4* ^−/−^ animals (Figure 6b, c, and Table 4). No differences between groups of female adult mice were observed (Table 4)

**TABLE 4:** Echocardiography parameters related to the “right ventricular” structure and function. Pulmonary blood flow parameters from young and adult, male and female, and wild‐type and *Trpm4* ^*−/−*^ mice.

		PV peak velocity (mm/s)	PV diameter (mm)	PV peak pressure (mm Hg)	mPAP (common) (mm Hg)
Young					
Wild‐type	♂	−708 ± 44 (*n* = 6)	1.498 ± 0.073 (*n* = 6)	2.02 ± 0.24 (*n* = 6)	75.90 ± 2.06 (*n* = 6)
*Trpm4* ^−/−^		−643 ± 31 (*n* = 5)	1.496 ± 0.062 (*n* = 5)	1.67 ± 0.15 (*n* = 5)	70.38 ± 1.82 (*n* = 5)
Wild‐type	♀	−605 ± 46 (*n* = 4)	1.327 ± 0.011 (*n* = 4)	1.48 ± 0.22 (*n* = 4)	72.38 ± 0.33 (*n* = 4)
*Trpm4* ^−/−^		−584 ± 21 (*n* = 4)	1.234 ± 0.106 (*n* = 4)	1.37 ± 0.10 (*n* = 4)	74.59 ± 2.15 (*n* = 4)
Adult					
Wild‐type	♂	−718 ± 15 (*n* = 10)	1.437 ± 0.085 (*n* = 10)	2.07 ± 0.08 (*n* = 10)	71.13 ± 0.33 (*n* = 10)
*Trpm4* ^−/−^		−605 ± 27 (*n* = 10)	1.411 ± 0.036 (*n* = 10)	1.49 ± 0.13 (*n* = 10)	71.09 ± 0.40 (*n* = 10)
Wild‐type	♀	−591 ± 17 (*n* = 8)	1.320 ± 0.052 (*n* = 8)	1.41 ± 0.08 (*n* = 8)	71.22 ± 0.62 (*n* = 8)
*Trpm4* ^−/−^		−562 ± 12 (*n* = 10)	1.445 ± 0.054 (*n* = 10)	1.27 ± 0.05 (*n* = 10)	70.21 ± 0.52 (*n* = 10)

*Note*: Values highlighted in dark gray (**p ⩽ 0.01) indicate a statistical difference between *Trpm4* ^−/−^ and wild‐type for the respective matching age and sex group.

### 3.5 Sex and aging influence the effects mediated by the knockdown of *Trpm4*

Taking the opportunity of the design experimental, we investigated the influence of sex on the effects observed by the decrease of expression of the TRPM4 protein.

The significant difference noticed for the pulmonary valve peak velocity between adult male and female wild‐type animals (average PV peak vel _wild‐type adult male_: −718 ± 15 mm/s (*n* = 10) and average PV peak vel _wild‐type adult female_: −591 ± 17 mm/s (*n* = 8); *p* < 0.0001) and the pulmonary valve peak pressure (average PV peak pressure _wild‐type adult male_: 2.07 ± 0.08 mm Hg (*n* = 10) and average PV peak pressure _wild‐type adult female_: 1.41 ± 0.08 mm Hg (*n* = 8); *p* < 0.0001)) was blunted in *Trpm4* ^−/−^ mice due to the alteration of those parameters in adult male *Trpm4* ^−/−^ mice (Table 4). Moreover, the low values for the HR (corresponding to a longer RR interval) measured in young and adult male *Trpm4* ^−/−^ mice (average HR _Trpm4−/− young male_: 460 ± 13 bpm (*n* = 13) and average HR _Trpm4−/− adult male_: 448 ± 12 bpm (*n* = 21) abolished the significant difference generally observed between sexes (Table 1).

By contrast, the decrease, during aging, of the left ventricle mass corrected in female *Trpm4* ^−/−^ mice leads to a significant difference of this parameter between adult male and female *Trpm4* ^−/−^ animals only (average LV mass corrected _Trpm4−/− adult male_: 128 ± 10 mg (n = 10) and average LV mass corrected _Trpm4−/− adult female_: 73 ± 8 mg (*n* = 10); *p* < 0.001) (Table 2). Moreover, linked to a broader QRS complex of the young female *Trpm4* ^−/−^ mice, this parameter significantly differs between young male and female *Trpm4* ^−/−^ mice compared to the respective wild‐type animals (average QRS _Trpm4−/− young male_: 9.8 ± 0.4 ms (*n* = 13) and average QRS _Trpm4−/− young female_: 11.2 ± 0.5 ms (*n* = 8); *p* < 0.05) (Table 1). Interestingly, the significant difference observed for the P duration between adult male and female wild‐type mice is abolished in adult *Trpm4* ^−/−^ mice due to the absence, in female *Trpm4* ^−/−^ mice, of an increase, during aging, of this parameter (Table 1). Finally, the weight taken by the animal during this study is significantly lower in female *Trpm4* ^−/−^ mice compared to the other groups (gain of body weight _wild‐type male_: 4 ± 2% (n = 18_young_/10 _adult_); gain of body weight _wild‐type female_: 30 ± 6% (*n* = 15 _young_ /10 _adult_); gain of body weight _Trpm4−/− male_: 9 ± 2% (*n* = 15 _young_ /11 _adult_); gain of body weight _Trpm4−/− fmale_: 12 ± 3% (*n* = 14 _young_ /10_adult_)).

### 3.6 The new TRPM4 inhibitor NBA affects atrioventricular conduction

**FIGURE 7:**
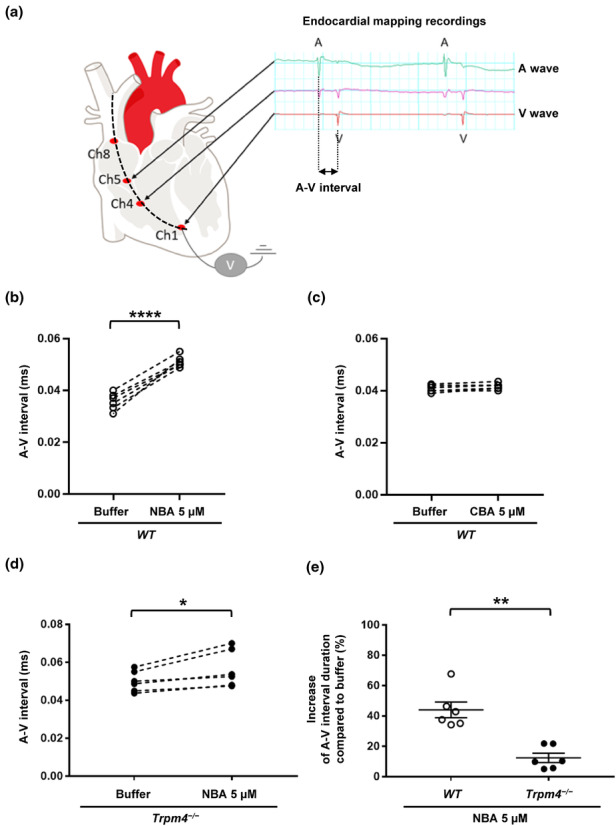
Effects of NBA and CBA on cardiac electrical activity in young male hearts using endocardial mapping approaches. (a) Schema showing the localization of the octopolar probe in the “right heart” to record the A and V waves using male wild‐type heart. The ECG recording shows the A wave (right atrial electrical activity) and V wave (right ventricle electrical activity). (b–d) Dot blots showing the evolution of the A‐V interval after the application of 5 μM NBA on wild‐type mouse hearts (b), 5 μM CBA on wild‐type mouse hearts (c), and 5 μM NBA on *Trpm4* ^−/−^ mouse hearts (d). (e) Dot plot summarizing the percentage of A‐V interval increase between wild‐type and *Trpm4* ^−/−^ mouse hearts after perfusion of 5 μM NBA. *p ⩽ 0.05, **p ⩽ 0.01, and ****p ⩽ 0.0001 (*n* ⩾ 4 per group). A paired nonparametric *t*‐test followed by a Wilcoxon matched‐pairs signed‐rank posttest was used to compare two paired groups (panels b–d). An unpaired nonparametric t‐test followed by a Mann–Whitney U posttest was used to compare two unpaired groups (panel e).

**FIGURE 8:**
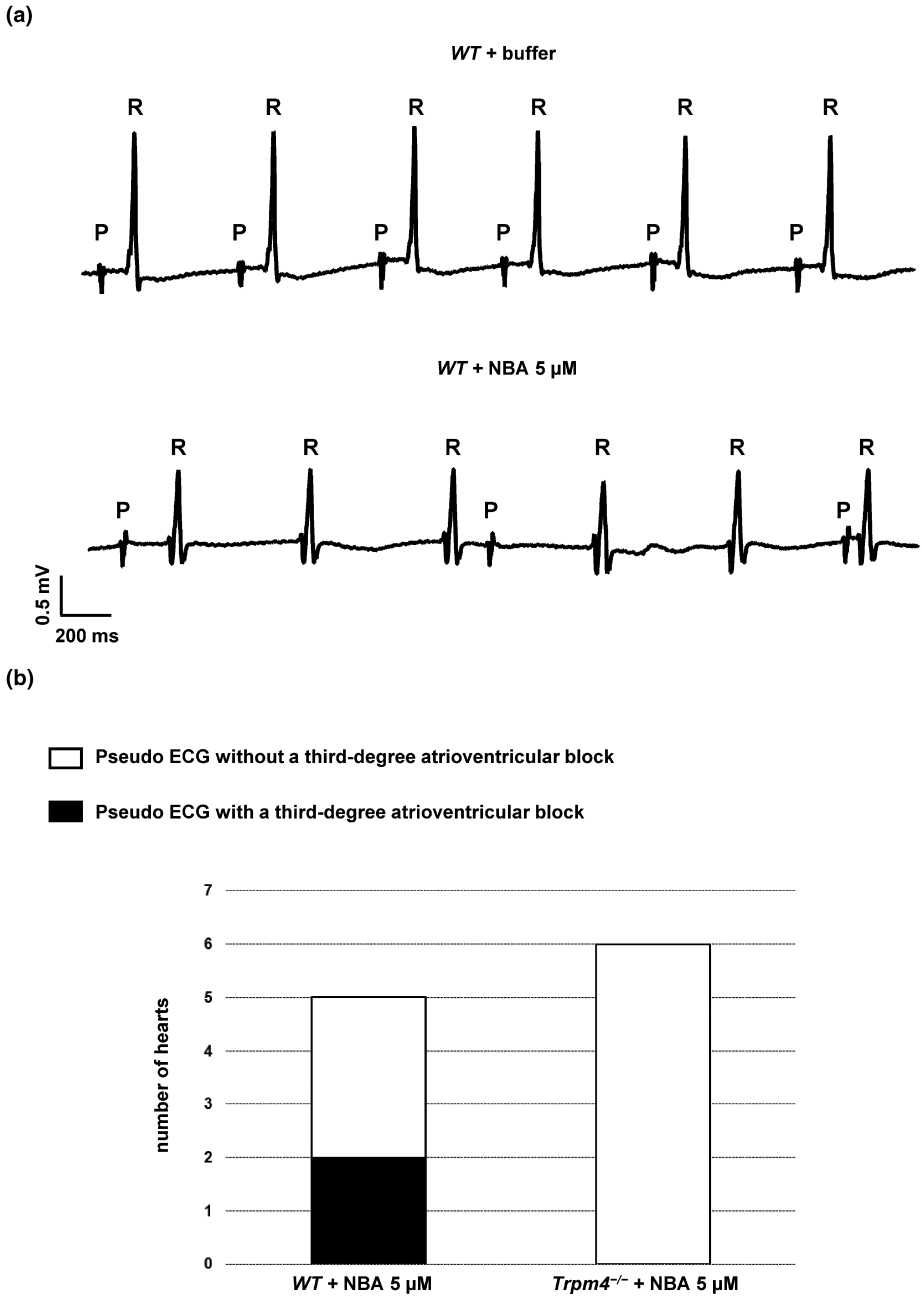
Perfusion of NBA leads to third‐degree atrioventricular blocks in wild‐type male mouse heart. (a) Representative ECG traces from pseudo‐ECG recorded before (top/buffer) and after (bottom/NBA 5 μM) NBA perfusion in wild‐type male mouse heart. P and R symbols represent the P wave and the QRS complex respectively. (b) Bar graph summarizing the number of pseudo‐ECG traces, from wild‐type and *Trpm4* ^−/−^ male mouse hearts, observed without (white) or with (black) a third‐degree atrioventricular block after NBA application.

We used the heart explanted‐Langendorff system to perform ex vivo ECG in isolated heart and endocardial mapping recordings in the absence and presence of NBA, a promising, recently described TRPM4 inhibitor (Arullampalam et al., 2021; Ozhathil et al., 2018). Knowing that the female *Trpm4* ^−/−^ animals did not display any electrical alteration and to reduce the number of animals used, this sex was omitted in these experiments (Table 1). Ex vivo ECGs did not show any HR differences between wild‐type and *Trpm4* ^−/−^ hearts (average HR _wild‐type adult male_: 208 ± 19 bpm (*n* = 6); average HR _*Trpm4*−/− adult male_: 224 ± 21 bpm (*n* = 6); ns) (Figure S3). Figure 7a shows representative endocardial traces recorded in wild‐type male mice hearts. The A and V waves correspond to electrical activation of the right atria and right ventricle, respectively, and are recorded at two different points of the octopolar probe (Ch5 and Ch1, respectively) (Figure 7a). The interval between A and V waves (A‐V interval) reflects the time required for electrical impulse propagation from the right atrium to the right ventricle through the atrioventricular node. Using A‐V interval as readouts for cardiac conduction, we investigated the effect of NBA on 12‐week‐old male wild‐type and *Trpm4* ^−/−^ hearts. As a control experiment, we investigated the potential effect of the vehicle (DMSO 0.05%) on the A‐V interval (Figure S4). As expected, no effect of the vehicle was observed on the A‐V interval (Figure S4). Perfusion of 5 μM NBA on young wild‐type mouse hearts significantly increased the A‐V interval (+44.0% ± 5.1%; *n* = 6) (Figure 7b). Five μM CBA, which inhibits human TRPM4 but not mouse TRPM4 in a heterologous expression system, did not modify the A‐V interval (Figure 7c) (Arullampalam et al., 2021). Unexpectedly, the A‐V interval in 12‐week‐old male *Trpm4* ^−/−^ mouse hearts was also slightly but significantly increased upon NBA application (+12.4% ± 3.1%; *n* = 6) suggesting that the NBA‐mediated A‐V conduction slowing in wild‐type heart does not completely depend on TRPM4 knockdown (Figure 7d). The increase of the A‐V interval is, however, still significantly higher in wild‐type than in *Trpm4* ^−/−^ hearts (Figure 7e). Surprisingly, the A‐V intervals under control perfusion seemed to be larger in *Trpm4* ^−/−^ than in wild‐type hearts (Figure 7b–d), however, further recording using more animals did not show any significant difference between wild‐type and *Trpm4* ^−/−^ A‐V intervals (Figure S5). Finally, pseudo‐ECG recordings analysis, on wild‐type male hearts revealed that the application 5 μM of NBA lead to a third‐degree atrioventricular block in 40% of cases (Figure 8a,b). No such effect was observed on *Trpm4* ^−/−^ male hearts (Figure 8b)

The slight but significant effect of NBA on the A‐V interval in *Trpm4* ^−/−^ mouse hearts (Figure 7c) suggests that NBA, at 5 μM, may have other targets than the TRPM4 channel. A recent study using canine left ventricle cardiomyocytes suggested that 10 μM CBA can inhibit the cardiac voltage‐gated sodium channel Na_v_1.5, which plays a key role in cardiac electrical impulse propagation (Dienes et al., 2021). We there for investigated whether NBA and CBA affect mouse Na_v_1.5, heterologously expressed in HEK‐293 cells, using the concentration applied to isolated murine hearts. A slight but significant decrease (−33.6% ± 6.8%; *n* = 8) of the sodium current was observed after the application of 5 μM NBA, but not with 5 μM CBA or the vehicle. This suggests that NBA, at this concentration, partially inhibits the voltage‐gated sodium channel Na_v_1.5 and may account for the increase of the A‐V interval shown in Figure 7c (Figure S6a,b)

## 4 DISCUSSION

Clinical studies have reported a strong link between *TRPM4* genetic variants and cardiac conduction disorders (Kecskes et al., 2015; Liu et al., 2013; Palladino et al., 2022; Stallmeyer et al., 2012). Experimental studies have revealed similar findings in mouse models (Demion et al., 2014; Guinamard et al., 2006; Liu et al., 2010; Mathar et al., 2010, 2014; Pironet et al., 2019) using different *Trpm4* ^−/−^ mouse models. However, investigations of cardiac function in *Trpm4* ^−/−^ mouse models reported heterogeneous results (Demion et al., 2014; Mathar et al., 2014; Vandewiele et al., 2022). In basal conditions, Demion et al. found that *Trpm4* ^−/−^ mice display multilevel conduction delays and arrhythmogenic activity (Demion et al., 2014). On the other hand, Mathar and colleagues found that *Trpm4* ^−/−^ hearts exhibited greater contractility in response to β‐adrenergic stimulation than wild‐types (Mathar et al., 2014), but they did not observe major cardiac alterations in absence of stress, which was also reported by Vandewiele and colleagues (Mathar et al., 2014; Vandewiele et al., 2022). It has been proposed that he discrepancies observed between these characterizations of *Trpm4* ^−/−^ were due to different genetic backgrounds (129/SvJ for Mathar et al., C57BL/6N for Vandewiele et al., and C57BL/6J for Demion et al.) (Medert et al., 2020). Based on these results, we generated a fourth *Trpm4* ^−/−^ mouse strain on a pure C57BL/6JRj background genetically close the one from Demion and colleagues, to increase our understanding of the role of TRPM4 under basal conditions. Importantly, we included female mice in our study, which were previously neglected.

Basic phenotyping characterization revealed that the body weight was 9.0% lower in adult male *Trpm4* ^−/−^ than in adult wild‐type animals. Surprisingly, Demion and colleagues noticed the opposite effect (male *Trpm4* ^−/−^ animals had higher body weight than wild‐type mice) (Demion et al., 2014). This could be explained by the effect of the genetic strain and substrain on metabolism (Ge et al., 2019; Siersbaek et al., 2020). Demion et al. did not provide further information concerning the substrain of the C57BL/6J mice.

In this study, we observed no major surface electrocardiogram alterations during the first 12 weeks of life in male or female wild‐type and *Trpm4* ^−/−^ mice. However, it is important to note that the anesthetic procedure can influence those parameters and it should be interesting to perform similar ECG recordings using telemetry approaches. The observation that the knockdown of TRPM4 did not affect the PR interval and QRS durations is not in line with what has been reported by Demion and colleagues (Demion et al., 2014). This can be explained by inter‐experimenter variability, since one must define the end of each P wave and the beginning of QRS complex manually, which are not trivial decisions. In addition, the genetic background (substrain) of the mice may also account for the observed differences between the two mouse models.

We performed echocardiography scans to identify any cardiac structural and hemodynamic alterations due to the deletion of the *Trpm4* gene. A slight increase in the left ventricular mass of male adult *Trpm4* ^−/−^ mice suggested left ventricle hypertrophy and potential dysfunction of the mitral valves. However, no systolic dysfunctions were observed in adult male and female animals. Interestingly, the parameters suggesting a mitral valve dysfunction were altered in young male *Trpm4* ^−/−^, but not in young female *Trpm4* ^−/−^. Similarly to our ECG data, these findings suggest that the onset of the cardiac alterations may be delayed in female animals. The reason remains yet to be elucidated. One potential mechanism may be that the diastolic dysfunction related to left ventricular hypertrophy causes mitral either a valve stenosis or regurgitation, which leads to a vicious circle and worsens diastolic function (Klein & Carroll, 2006). The increase of NT‐proBNP observed in *Trpm4* ^−/−^ compared to their respective controls supports the ventricular hypertrophy observed during echocardiography in male mice. However, we did not investigate whether this hypertrophy may be related to hyperplasia, as observed by Demion and colleagues (Demion et al., 2014). In female animals, this increase suggests that the absence of TRPM4 protein may lead to increased ventricular wall stress in female hearts, without overt structural alteration of the cardiac tissue. The increase in NT‐proBNP could also be related to other noncardiac diseases, such as nephropathies. Indeed, TRPM4 was shown to be expressed in the kidney (Flannery et al., 2015; Tsai et al., 2010). More experiments are needed to better understand why *Trpm4* ^−/−^ adult female mice present higher NT‐proBNP concentration than wild‐type female animals.

This study assessed the “right ventricular” systolic function of *Trpm4* ^−/−^ mice for the first time. Adult *Trpm4* ^−/−^ male mice, but not female, showed significant alterations in their right ventricular systolic function. When considering the etiology of this dysfunction, we propose two mechanisms. On one hand, *Trpm4* gene deletion could directly impact systolic dysfunction through a cardiomyocyte‐mediated effect, or on the other hand, the gene deletion may indirectly impact right ventricular function by affecting the mitral valve, leading to stenosis or regurgitation, which increases pulmonary artery pressure. Those potential dysfunctions should be investigated using histologically approaches.

The use of male and female animals at different ages in this study brought forward interesting new findings. Mainly, adult male *Trpm4* ^−/−^ showed cardiac alterations. The absence of dysfunctions at a young age may be explained by the fact that the respective cardiac alterations take time to develop, as it is in humans, where hypertrophic cardiomyopathy is a hallmark of cardiac aging. The dynamic expression of TRPM4 protein may also be related to this time‐dependent cardiac alteration. In mouse CA1 pyramidal neurons the expression of TRPM4 has been shown to increase after birth (Riquelme et al., 2021), while it decreases with age in urinary bladder smooth muscle cells (Maxwell et al., 2021). Additionally, a cyclic expression of TRPM4 has been shown in female vomeronasal neurons due to the ovarian sex hormone cycle (Eckstein et al., 2020). Overall, these data suggest that the expression pattern of TRPM4 protein over time is highly regulated allowing TRPM4‐expressing organs to properly function. Its expression at adult age may help to prevent cardiac disorders. Finally, environmental age‐dependent triggers may also explain these TRPM4‐dependent alterations in the heart. Female *Trpm4* ^−/−^ mice did not display any cardiac alterations in this study, which does not exclude that they may develop them later in life. Sex hormones might protect females from cardiac disorders, as already suggested in previous publications (Iorga et al., 2017). It is worth noting that the prevalence of cardiac disorders linked to TRPM4 dysfunction (e.g., Progressive familial heart block type I) has been suggested to be more prevalent in males than in females, as observed in this study (Ehdaie et al., 2018).

Interestingly, endocardial recordings using the new potent TRPM4 inhibitor NBA suggest that TRPM4 inhibition leads to atrioventricular conduction slowing and/or atrioventricular block in wild‐type hearts as already reported by Demion and colleagues (Demion et al., 2014). It is worth noting that the in vivo surface ECGs in male *Trpm4* ^−/−^ mice did not show any alteration of the atrioventricular conduction. However, this interval reflects the time required for the electrical stimulus to travel through all the atria and ventricles. In the endocardial mapping, the recording leads have been localized only in the “right heart,” while the electrode recording the electrical activity of the “right atrium” is close to the atrioventricular node. This difference may explain the noted discrepancy. In addition, an important element which differs between these two models is the absence of regulation by the autonomic nervous system (ANS) in the endocardial mapping experiments. In vivo, the ANS may mediate a compensatory mechanism, masking slowing of the atrioventricular conduction. This remains to be investigated.

Finally, the observations from endocardial mappings using NBA (acutely inhibiting TRPM4) do not perfectly resemble those in the *Trpm4* ^−/−^ mouse model, in which TRPM4 is not inhibited but absent. The constitutive TRPM4 knockdown may induce compensatory effects during development explaining these different observations. Nevertheless, this data supports the previous findings from heterologous expression systems suggesting that NBA may be an ideal compound to investigate the role of TRPM4 in the heart (Arullampalam et al., 2021). However, as shown in this study, NBA may also have off‐target effects (e.g., on the cardiac voltage‐gated sodium channel Na_v_1.5 channel). All results presented in this study should thus be interpreted carefully, considering that the Na_v_1.5‐mediated sodium current has been shown to be decreased in this mouse model (Ozhathil et al., 2021). The cardiac alterations observed in *Trpm4* ^−/−^ mice may thus not only be due to the knockdown of TRPM4 protein but may also be mediated by Na_v_1.5 dysfunction via either its downregulation (decrease of Na_v_1.5 current) and/or its inhibition (decrease of Na_v_1.5 current via NBA). Interestingly, a recent publication showed that meclofenamate, a new TRPM4 antagonist structurally close to NBA, also blocks Na_v_1.5 current, suggesting that compounds with a structure similar to NBA such as meclofenamate may not only inhibit TRPM4 but also Na_v_1.5 channels (Vandewiele et al., 2022). Given that (1) the NBA concentration used in this study is 20 times higher than the IC_50_ determined to inhibit mouse TRPM4 in a heterologous expression system (NBA‐IC_50‐TRPM4_ = 0.215 μM) (Arullampalam et al., 2021) and (2) the decrease of mouse Na_v_1.5 current, using 5 μM NBA is small, investigating the effect of NBA on Na_v_1.5 current in depth will be of interest. An important step will be to perform dose–effect response and other protocols to study the influence of biophysical parameters on this inhibition (e.g., variation of the resting membrane potential, the frequency of stimulation, and the influence of β‐subunits coexpression). Such investigations will provide important information on optimal NBA concentrations to be used in similar future experiments, to avoid effects mediated by Na_v_1.5 current.

## 5 LIMITATIONS

Among the limitations of this study, it is worth noting that TRPM4 is widely expressed in different organs and tissues, including the brain and the endocrine system. The cardiac dysfunctions observed after constitutive *Trpm4* gene deletion may be due to indirect mechanisms or related to a compensatory mechanism. Similar experiments using inducible *Trpm4* ^−/−^ and/or cardiac‐specific knockdown mouse models may characterize the role of TRPM4 in cardiac function in a more specific manner.

It has proven challenging to reproduce data using similar *Trpm4* ^−/−^ mouse models, as illustrated by the respective publication (Demion et al., 2014). The strain and substrain hypotheses are relevant but may not explain all the observed discrepancies. The techniques used to characterize the models are complex and are prone to experimenter‐dependent results. Furthermore, the Langendorff approach used in this publication does not allow us to investigate whether the autonomic nervous and/or hormonal systems affect TRPM4‐dependent cardiac regulation. Overall, based on our data and those from previous researches, we suggest that close inter‐institutional and ‐national collaboration will be crucial in setting up and standardizing the techniques to achieve reproducible results regarding the role of TRPM4 in cardiac tissue (Demion et al., 2014; Mathar et al., 2014; Vandewiele et al., 2022).

Another obvious limitation of this investigation is that the knockdown of TRPM4 protein in this mouse model leads to a decrease of the Na_v_1.5‐mediated sodium current (Ozhathil et al., 2021). Moreover, since other voltage‐gated sodium channel isoforms are also present in cardiac cells (including those from the conduction system), this TRPM4 knockdown may affect other Na_v_ isoforms, too, which may have implications for the results obtained with this mouse model. In addition, Vandewiele and colleagues used another *Trpm4* ^−/−^ mouse model to investigate the effect of knocking down TRPM4 expression on Na_v_1.5 current (Vandewiele et al., 2022). Interestingly, they did not find any alterations in Na_v_1.5‐mediated sodium current, suggesting that the effect observed in voltage‐gated sodium current may also be (sub)strain specific (Vandewiele et al., 2022). Overall, the results presented in different publications using different *Trpm4* ^−/−^ mouse models should be interpreted with caution.

## 6 CONCLUSIONS

In conclusion, the cardiac investigation of this new *Trpm4* ^−/−^ mouse model confirms that TRPM4 plays a role in the electrical function of the heart in an age‐dependent manner. This study also reveals significant differences between male and female animals that have never been reported before. In addition, the investigation of the effects of NBA on heart function highlights the direct or indirect role of TRPM4 in the atrioventricular node. However, the discrepancies between the results presented in this study and those from previous publications suggest that the investigation of the role of TRPM4 in the heart in the absence of cardiac stress/dysfunction may be influenced by many unidentified factors, such as mouse substrain and hormones, rendering its investigation under these conditions challenging.

## AUTHOR CONTRIBUTIONS

PA, MCE, and JSR conceived and designed the experiments. PA, MCE, MB, SG, and JSR collected, analyzed, and interpreted the data. PA, JSR and HA drafted the manuscript.

## FUNDING INFORMATION

This work was supported by the The National Centres of Competence in Research (NCCR) TransCure (grant no. 51NF40‐185,544 to HA) and the Swiss National Science Foundation (grant no. 310030_184783 to HA).

## CONFLICT OF INTEREST STATEMENT

All other authors declare no competing interests.

## Supporting information


Figure S1

Figure S2

Figure S3

Figure S4

Figure S5

Figure S6

## Data Availability

The data that supports the findings of this study are available in the supplementary material of this article and are available on request from the corresponding author.
